# Cross-Platform Microarray Meta-Analysis for the Mouse Jejunum Selects Novel Reference Genes with Highly Uniform Levels of Expression

**DOI:** 10.1371/journal.pone.0063125

**Published:** 2013-05-09

**Authors:** Florian R. L. Meyer, Heinrich Grausgruber, Claudia Binter, Georg E. Mair, Christian Guelly, Claus Vogl, Ralf Steinborn

**Affiliations:** 1 Genomics Core Facility, VetCore, University of Veterinary Medicine, Vienna, Austria; 2 Institute of Anatomy, Histology and Embryology, University of Veterinary Medicine, Vienna, Austria; 3 Department of Crop Sciences, University of Natural Resources and Life Sciences, Tulln, Austria; 4 Institute of Animal Nutrition and Functional Plant Compounds, University of Veterinary Medicine, Vienna, Austria; 5 Center for Medical Research, Medical University of Graz, Graz, Austria; 6 Institute of Animal Breeding and Genetics, University of Veterinary Medicine, Vienna, Austria; Northwestern University Feinberg School of Medicine, United States of America

## Abstract

Reference genes (RGs) with uniform expression are used for normalization of reverse transcription quantitative PCR (RT-qPCR) data. Their optimization for a specific biological context, *e.g.* a specific tissue, has been increasingly considered. In this article, we compare RGs identified by expression data meta-analysis restricted to the context tissue, the jejunum of *Mus musculus domesticus*, i) to traditional RGs, ii) to expressed interspersed repeated DNA elements, and iii) to RGs identified by meta-analysis of expression data from diverse tissues and conditions. To select the set of candidate RGs, we developed a novel protocol for the cross-platform meta-analysis of microarray data. The expression stability of twenty-four putative RGs was analysed by RT-qPCR in at least 14 jejunum samples of the mouse strains C57Bl/6N, CD1, and OF1. Across strains, the levels of expression of the novel RGs *Plekha7*, *Zfx*, and *Ube2v1* as well as of *Oaz1* varied less than two-fold irrespective of genotype, sex or their combination. The gene set consisting of *Plekha7* and *Oaz1* showed superior expression stability analysed with the tool RefFinder. The novel RGs are functionally diverse. This facilitates expression studies over a wide range of conditions. The highly uniform expression of the optimized RGs in the jejunum points towards their involvement in tightly regulated pathways in this tissue. We also applied our novel protocol of cross-microarray platform meta-analysis to the identification of RGs in the duodenum, the ileum and the entire small intestine. The selection of RGs with improved expression stability in a specific biological context can reduce the number of RGs for the normalization step of RT-qPCR expression analysis, thus reducing the number of samples and experimental costs.

## Introduction

RT-qPCR is considered the gold standard for measuring the number of copies of specific cDNA targets in a sample. The quality of RT-qPCR expression data strongly depends on accurate transcript normalization. This is usually accomplished using reference genes (RGs). Ideally, the steady-state transcript level of a RG is constant in all samples under investigation, regardless of tissue type, physiological state, genotype or experimental condition. Even subtle variations in RG expression can have a marked influence on the outcome of experiments, especially when the target gene shows a modest change in transcript abundance [1]. A proper normalization strategy is therefore among the key elements in the Minimum Information for Publication of Quantitative Real-Time PCR Experiments (MIQE) guidelines [2] since even a relatively modest two-fold change in expression of a target gene may cause dramatic biological effects [3], [4]. Validation of each RG on an individual basis for all treatment and experimental conditions is paramount for accurate data interpretation when normalizing for inter-sample variability in RT-qPCR experiments. A threshold criterion of Δ*Cq* ≤ ±0.5 was recommended for RG suitability, since fluctuations of this magnitude may be expected via technical variation alone [1]. The current trend is to first select candidate RGs (*e.g.* ≥8; [5]), then to assess their expression uniformity in all samples of the experiment and finally to use a suitable subset for RT-qPCR normalization [6]. Expression stability can be assessed by software tools such as GeNorm [6], Global Pattern Recognition [7], NormFinder [8], BestKeeper [9] or Equivalence test [10] or by a stability index based on the analysis of variance (ANOVA) model [11]. RG candidates can be ranked by the integrated web-based comprehensive tool RefFinder (http://www.leonxie.com/referencegene.php; [12]) compensating for the individual weaknesses of the major computational programs geNorm, Normfinder, BestKeeper, and the comparative Δ*Cq* method [13]. Candidates for this approach can be pre-selected from traditional RGs (tRGs) such as *Gapdh*, *Actb* and *Hprt*. RGs of this class have been used in Northern blotting, RNase protection and conventional RT-PCR experiments over many years [14], but are still being widely used for RT-qPCR normalization. The class of non-protein-coding RNAs represents another pool of genes to select from [15].

Interplatform microarray analysis is an increasingly important research tool for expression analysis of mRNA (reviewed in [16]) and miRNA [17]. Its importance is also indicated by the availability of open source repositories for cross-platform microarray data analysis [18]. Nevertheless external validation of gene expression by RT-qPCR frequently failed to confirm an expression pattern predicted by cross-platform meta-analysis, probably due to heterogeneous sample cohorts and discrepancy of probe design and experimental protocols. Sophisticated normalization techniques have been developed to solve or alleviate the problem [16]. Meta-analysis of genome-wide expressed sequence tags (EST), serial analysis of gene expression (SAGE) and/or microarray gene expression data that can be collected from public sources [19], [20], [21], [22], [23] has helped ensure that the selection of RGs is no longer based on educated guesswork and intuition. Expression meta-analysis of various tissues, cell lines and conditions has resulted in the identification of more universal RGs such as *Oaz1* and several ribosomal protein genes showing high stability across a multitude of cell types and experimental conditions [20], [24]. Members of this class are termed universal RGs (uRGs).

Analogous to the global normalization of RNA expression microarrays, RT-qPCR data can be normalized by a pooled signal from multiple random genes obtained by priming with an arbitrary, short oligonucleotide [25]. Expressed interspersed repetitive elements, a redundant and diverse set of expressed mobile DNA sequences, have also been used in the hope to find a universal normalization strategy for all biological sample types and experimental conditions [26]. We will refer to such a gene as “repeat element RG” (rRG). They are recommended for the normalization of minute amounts of RNA based on high expression levels [26] and are less stimulus dependent as a result of transcription from diverse genomic regions.

In diverse biological contexts normalization of RT-qPCR data was improved by selecting RGs based on expression meta-analysis ([27], [28], [29], Table S1). We will refer to such a gene as “meta-analysed RG” (mRG). A gene of this class fulfils the criterion of uniform expression, and was selected based on context-restricted meta-analysis and not just by educated guesswork. For mRG selection, transcriptome data collected either by sequencing or array-based technologies should be mined. When optimization of RGs for a specific context tissue is the matter [30], [31], [32], [33], a similar approach can be devised. The selection of RGs specific for a particular context (*e.g.* a tissue) reflects that currently no gene exhibiting a stable pattern of expression across all conditions and in all tissues is known [33]. As only few genes are uniformly expressed across a wide range of conditions, restriction to a specific context offers more candidate genes for RG selection, and thus genes involved in more pathways.

The more RGs are used the higher the costs and the more cDNA is needed. Hence lowering the number of RGs lowers the costs, especially when many samples are needed. RG optimization is crucial for high precision measurement required when differences among groups are small [34].

In this study, the mouse jejunum was chosen as the biological context. The jejunum has a central role in the digestion and absorption of nutrients. It is a key tissue in 90-day rodent-feeding studies and in Reproductive Assessment by Continuous Breeding design [35], where chronic effects of subclinical infections, or the long-term incorporation of dietetic or potentially toxic compounds are studied. With a meta-analysis restricted to the mouse jejunum, we identified mRGs with highly uniform RNA levels. In brief, the expression data crossed comprised publicly available and internal microarray data of different microarray platforms based on cDNA, or on 60-mer or 25-mer oligonucleotide probes. The expression uniformity of the selected mRGs was analysed by RT-qPCR in samples of inbred and outbred mice using members of the tRG, uRG and rRG classes for comparison.

## Results

**Figure 1:**
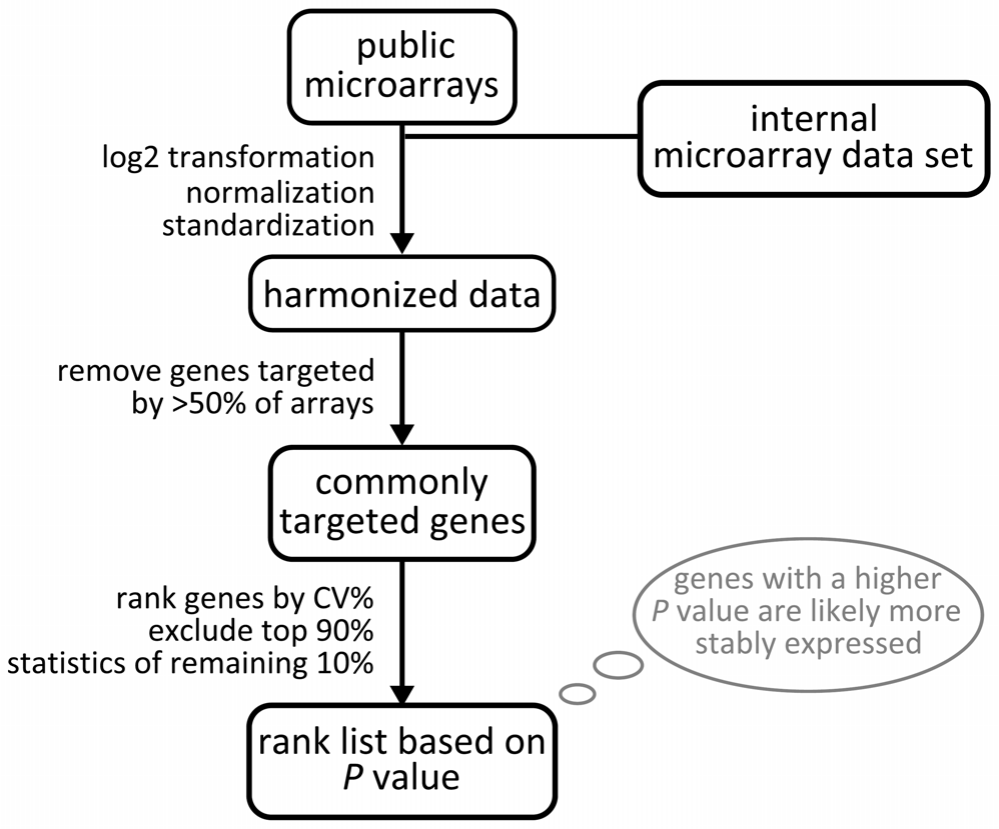
Algorithm for meta-analysis of multiplatform microarray expression data to identify mRGs optimized for a context tissue.

We performed a meta-analysis of microarray expression data restricted to a specific target tissue- or tissue-section to derive novel mRGs with high expression stability and from a wide variety of cellular processes. Because of the small number of single-platform microarrays available for the selected tissue, *i*.*e*. the jejunal segment of the small intestine, a protocol for the meta-analysis of data from different microarray technologies was developed (Figure 1). Briefly, technologically different microarray expression datasets were integrated with platform-specific calibration methods, followed by the removal of genes not targeted by the majority of platforms. Only the 10% of genes whose expression was most uniform, as assessed by ranking their coefficient of variation in percent (CV%) values, were selected for statistical evaluation. We selected the genes with little variation assuming that differentially regulated genes will have high variation, while putative RGs will have little. In particular, genes with the highest *P*-values were selected as the most likely candidate mRGs.

The novel pipeline was used for meta-analysis of jejunal RNA expression data from 20 external studies and one internal dataset comprising a total of 53 jejunal microarrays (Table S2). They were derived from the following nine array platforms: six Mouse Genome 430A 2.0 Arrays (Affymetrix), five IncyteMouseGEM1 Arrays (non commercial), twenty four Mouse Genome 430A Arrays (Affymetrix), nine MG-U74A Arrays (Affymetrix), three MG-U74B Arrays (Affymetrix), three MG-U74C Arrays (Affymetrix), two MG-U74Av2 Arrays (Affymetrix) and nine Gene Expression Array System Arrays (Applied Biosystems). Arrays used cDNA, 60-mer or 25-mer oligonucleotide probes. Signals were generated by chemoluminescence, colorimetry or by one or two fluorophores.

Next, the expression stability of the selected RG candidates was measured by RT-qPCR in inbred (C57Bl/6N) and outbred (CD1 and OF1) mice.

### Meta-Analysis Based on *P-*Value Statistic (Protocol I)

A list of ‘top 100’ mRG candidates was generated by this meta-analysis protocol (Table S3). From this list 21 genes were randomly selected for RT-qPCR validation (Table 1). Variation in expression of the novel mRGs was investigated in at least 14 jejunum samples derived from one common inbred (C57Bl/6N, n ≥4) and two outbred (OF1 and CD1, n ≥10) strains. For comparison we measured the stability of expression *i)* of the tRG class gene *Hprt* still being widely used in parasitic infections studies [36], *ii)* of the B1 and B2 elements belonging to the short interspersed nuclear element (SINE) family and representing the rRG class and *iii)* of the uRG class members *Rps29*, *Rpl4* and *Oaz1* [24]). *Oaz1*, a weakly expressed gene [20], was recommended by two meta-analyses of human and/or mouse expression data from a wide range of conditions [20], [24]. These studies, however, did not address inter-sample variation between biological and technical replicates for a single context type.

**Table 1:** Rank of mRGs based on their fold-change differences in expression measured by RT-qPCR.

Rank^1^	Gene symbol	Median *Cq* 2	Fold change^3^	Function
Meta-analysis I				
1 (22)	*Plekha7*	22	1.7	biogenesis and maintenance of adherents junctions
2 (11)	*Zfx*	24	1.7	transcription factor
3 (29)	*Ube2v1*	22	1.9	polyubiquitination
4 (6)	*Tom1*	20	2.0	intracellular trafficking
5 (71)	*Tmem14c*	20	2.0 (2.7)	heme biosynthesis
6 (19)	*Hjurp*	23	2.1	centromer formation
7 (9)	*Cxx1b*	27	2.1	unknown (retrotransposon derived protein)
8 (5)	*D15Ertd30e*	25	2.1	unknown
9 (12)	*Tspan15*	22	2.1	member of tetraspanin family
10 (36)	*Zfyve19*	24	2.1	DNA repair
11 (28)	*AI314976*	20	2.1 (3.9)	tRNA splicing
12 (50)	*Hadhb*	20	2.3 (4.7)	fatty acid metabolism in mitochondria
13 (17)	*Zfp598*	22	2.3	Zink finger protein
14 (37)	*Atp6v0d1*	22	2.4	H^+^ transmembrane transporter activity
15 (1)	Pcdha@	26	2.6	cell adhesion
16 (83)	*Fbln1*	18	2.6	extracellular matrix organisation
17 (67)	*Gsr*	20	2.8 (4.1)	cellular antioxidant defence
18 (3)	Gag@4	20	3.3	retroviral group-specific antigen
19 (52)	*Slc52a3*	20	4.0	riboflavin transport
20 (86)	*B3gnt3*	22	4.1	protein amino acid glycosylation
21 (4)	*Fcer2a*	29	9.5	nitric oxide biosynthesis
Meta-analysis II				
1 (5)	*Aldoa*	21	3.6	glycolysis and gluconeogenesis
2 (2)	*Usmg2*	28	12.4	unknown
3 (1)	*St6galnac1*	31	>53.2^5^	protein glycosylation

@: gene cluster.

1 Rank of RT-qPCR followed by rank of meta-analysis in brackets.

2 *Cq* at 100% amplification efficiency.

3 the value for the fold-change range given in brackets includes outlier sample.

4 *Gag* genes cross hybridizing to the withdrawn *1200016E24Rik* sequence: *Gm4268*, *Gm3817*, *Gm4569*, and/or *Gm2251.*

5 no amplification signal for one qPCR replicate and one cDNA due to limit of detection.

**Figure 2:**
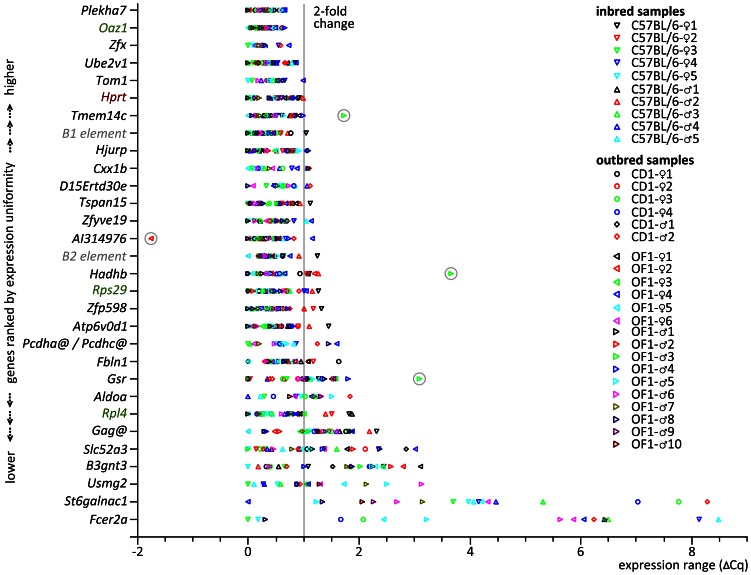
Novel mRGs selected for the mouse jejunum show high uniformity of expression in RT-qPCR. For each gene the Δ*Cq* range of ten outbred and four inbred samples was determined and used as a ranking criterion for the list of mRGs. Individual *Cq*s were corrected to 100% amplification efficiency and plotted as difference to the sample with the lowest *Cq*. Outliers with higher (*AI314976*) and lower transcript expression (*Tmem14c*, *Hadhb*, and *Gsr*) are encircled. RG classes are depicted by colour: tRG (brown), uRG (green), rRG (grey) and mRG (black). The line depicts the delimiter range of ≤ two-fold expression variation for a RG. *@*: gene cluster.

A list of candidate RGs ranked in order of their stability in RT-qPCR is presented in Table 1. As expected there is no complete match with the ranking obtained from our novel tissue-restricted meta-analysis. This can be explained by the technological inconsistency of the two methods [37] and the differences in the biological conditions and the genetic background of the mice models used. Notably, two of the three uRGs selected for comparison in RT-qPCR, *Oaz1* and *Rps29*, were absent from the top 10% of genes selected for their low CV%. This may mean that our 10% cut-off was too restrictive as both genes were uniformly expressed according to RT-qPCR. This does not contradict our selection strategy considering that hundreds of genes show low expression alteration, *i.e.* are putatively suitable for RT-qPCR normalization. In detail, the expression variance of ≦2-fold determined for four novel mRGs was lower (*Plekha7*) or comparable (*Zfx, Ube2v1*, *Tom1*) to that of the best (*Oaz1*) of the three uRGs tested and to the tRG class member *Hprt* (Figure 2). A similarly low variance was observed for *Tmem14c* except of a single OF1 outlier sample. A slightly wider range of expression variation was determined for the B1 and B2 elements, members of the rRG class (Figure 2).

To clarify cross-hybridization to the sequence *1200016E24Rik* (meta-analysis rank 3 in Table 1) which was withdrawn from NCBI in 2010, we used fluorescent dye terminator Sanger sequencing to determine the sequence of the amplicon produced by RT-qPCR. A basic local alignment search using Blastn identified complete homology to predicted mRNAs encoded by *Gag* genes located on chromosomes 1, 7, 10 and 12. The sequence was submitted to the GenBank under the accession number HQ681193.

The genes in the novel mRG set optimized for use in jejunum samples are involved in a variety of biological processes regulating a wide range of cellular functions (Table 1). This is also reflected by the fact that 18 of our mRGs were assigned to 128 unique categories of gene ontology terms (data not shown). Thus, for multigene normalization of RT-qPCR expression studies the novel set of mRGs provides extremely high flexibility with regard to experimental stimulus and condition.

### Application of the Tissue-Restricted Meta-Analysis Approach to the Duodenum, the Ileum and the Small Intestine

Considering the identification of highly uniformly expressed genes in the mouse jejunum (see above), the meta-analysis protocol I was applied to the other two sections of the small intestine, the duodenum and the ileum, and to the microarray data set of small intestine samples. In the case of the small intestine, the expression dataset used for the meta-analysis comprised 22 studies with a total of 220 microarrays derived from samples of defined (duodenum: n = 23, jejunum: n = 53, ileum: n = 63) and unspecified (n = 81) origin. The resulting three ‘top 100’ lists of genes from the section/tissue-restricted meta-analyses are listed in Table S4.

The rank lists obtained by section-specific meta-analyses for the jejunum and the ileum contained a similar number of genes above the significance threshold (*P* = 0.05; 10 or 14, respectively). The finding that none of the 15 genes ranked top in the jejunum appeared among the top 100 of the ileum data is in line with our concept of mRG optimization for a specific tissue/section considering that there is a large number of stable transcripts to select from [33].

In contrast, in the meta-analysis performed for the duodenum, ten times more genes were above the level of significance (n = 123). This is probably caused by impaired precision of measurement, as duodenal samples are rich in RNases, as is the adjacent pancreas. Likewise, none of the 15 genes ranked top in the duodenum was among the top 100 of the jejunum or ileum data.

**Figure 3:**
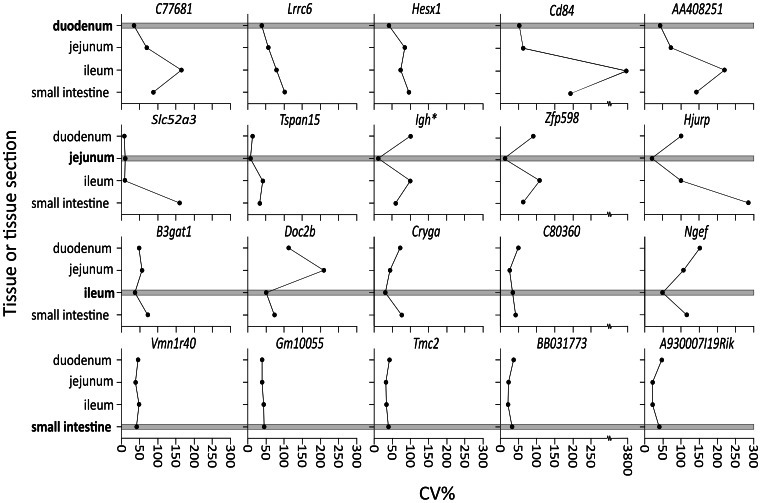
Meta-analyis of microarray data restricted to the context tissue/tissue section (small intestine or its sections) selects mRGs with low expression variability. This is indicated by a low CV% of a mRG identified as top ranking by a restricted meta-analysis in a specific context tissue/section (boxes) but a higher CV% for the same mRG in a non-context tissue/section. *Probe also detects *Igha* and *Igh-VJ558.*

To illustrate the reduced variation of expression of the genes found by this restricted meta-analysis approach, the CV%s of the five top-ranking mRGs of each section/tissue list were compared reciprocally (Figure 3). In most cases the comparison yielded the expected pattern of a low CV% for the context section of the restricted meta-analysis and elevated CV%s for the non-context sections/tissue.

In summary, the meta-analyses performed for RNA expression data for the jejunum, the duodenum, the ileum and the small intestine as the selected context tissue provided lists, of which we reported the top 100 candidates for mRGs. The novel mRGs had not been previously found by other meta-analyses of the protein-encoding transcriptome across a wide range of tissues and experimental conditions [20], [24].

### Meta-Analysis Protocol Normalizing for Differences in Array Number per Study (Protocol II)

Six of the eight studies (75%) and 39 of the 53 arrays (73.6%) considered in our intermicroarray platform meta-analysis for the jejunum used Affymetrix short oligomer microarrays (Table S2). It was reported that the Affymetrix microarray platform may lead to the detection of excessive numbers of false positives [38]. Considering that more than two thirds of the chips in the meta-analysis were of this type, we therefore asked whether our data integration approach could be affected by this phenomenon. We assumed that a normalization step attributing equal weight to an experimental study irrespective of the number of arrays used would diminish the impact of studies with high array numbers and would reduce bias by an individual biological context, thus resulting in an enrichment of false positive hits among the top ranking genes. In other words, we assumed that this normalization procedure would enrich for artefacts related to the Affymetrix short oligomer microarray technology.

From the resulting list obtained by this protocol in which each experimental study contributed equally irrespective of differences in microarray number we tested six genes with top *P*-values for expression in RT-qPCR (Table S5). Not surprisingly, we found two genes that were not expressed irrespective of whether the primer targeted an exonic site or the 3′ untranslated region (*BC147527*, *AI594671*; Table S5) using an equimolar mixture of jejunal cDNAs from inbred and outbred samples as template. We regarded these two cases as false positive microarray expressions considering that they resulted exclusively from contributions of Affymetrix microarrays and that the same mouse strain was used for microarray and RT-qPCR analysis (Table S2 and Material and methods section). Moreover, the gene *Gm14743* showed expression close to the lower end of the quantitative dynamic range.

The target sequences of the Affymetrix probe sets of the three genes *BC147527*, *AI594671* and *Gm14743* did not contain G-spots, *i.e.* sequences of four or more guanines. This excludes that unspecific hybridization resulted from a local reduction in probe density mediated by adjacent G-quadruplex formation via four G-spot probes [38]. It remains to be seen whether advances in the normalization procedure of microarray expression data can reduce the number of false positives. For example, significantly fewer false positives can be obtained from transcripts of low abundance if the broadly accepted Affymetrix MAS5 normalization algorithm (Table S2 and this study) is replaced by a combination of intra-experiment Robust Multi-array Average (RMA) normalization and inter-experiment correction. This change in the processing of Affymetrix DNA microarray expression data derived from multiple studies was recently introduced by the expression meta-analysis tool Genevestigator (www.genevestigator.com), a database and Web-browser data mining interface for Affymetrix GeneChip data [19].

The other three top-ranking genes selected for expression analysis by RT-qPCR (*St6galnac1*, *Usmg2*, and *Aldoa)* showed signals within the dynamic range in the pooled cDNA template. Their expression was subsequently tested on individual samples *(* Figure 2). The high expression variation of *St6galnac1* (Δ*Cq* range of 8.3 corresponding to a 311-fold change) is most likely just another indication of the inconsistencies between microarray expression data analysis and our RT-qPCR study regarding biological material and/or principle of detection method.

In summary, irrespective of the false-positive cases in the top of the gene list obtained by the weighted meta-analysis, the selected candidate RGs are worth further validation. RT-qPCR-based expression profiling of a larger set of candidate genes, *e.g.* including genes identified by microarray platforms using short and long oligonucleotide probes (Affymetrix and Applied Biosystems microarray platforms, respectively; genes with ranks ≥24 in Table S5), could potentially add valuable information on tissue-optimized RGs. However, for financial reasons this was not pursued in the present study. The following results only refer to mRGs identified by meta-analysis according to protocol I.

### Genes Stably Expressed Irrespective of Strain, Sex, Strain by Sex Interaction, and Random Effect

**Figure 4:**
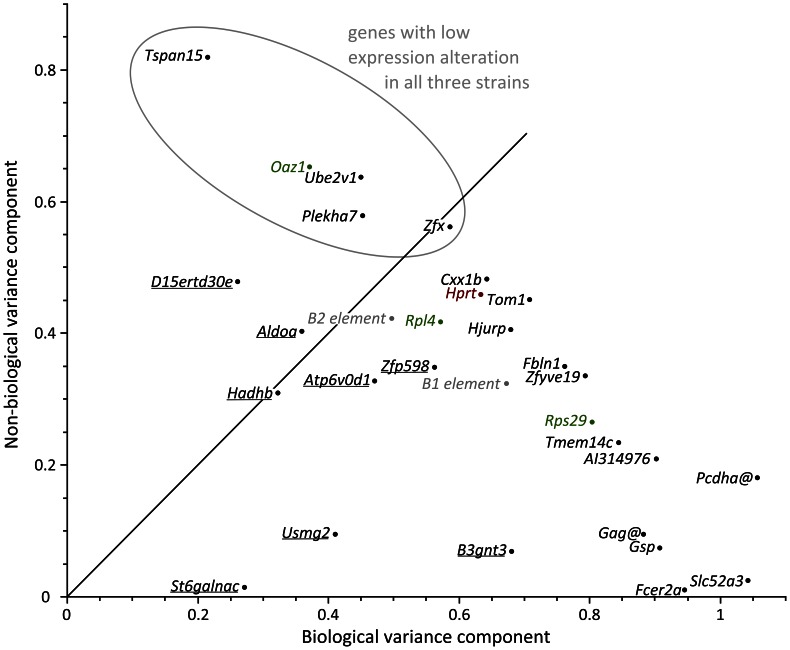
The general linear mixed model analysis of variance components of random effects (mouse individual, residual non-biological error) identified a subset of mRGs with low variation in expression irrespective of fixed effects such as strain, sex or strain by sex, and the random effect of mouse individual. Genes underlined showed a significant strain effect. For genes not underlined there was no significant fixed effect (strain, sex, strain by sex). For genes above or near the diagonal, the proportion of the non-biological error (duplicate qPCR reaction and repetition of experiment with another set of mice) was lower than or similar to the level of biological variation (individual mice). RG classes are depicted by colour: tRG (brown), uRG (green), rRG (grey) and novel mRGs selected for the context tissue of this study (black). *@*: gene cluster.

We analysed whether the expression of the novel jejunal mRGs is dependent on strain, sex or strain-by-sex interaction and assessed the random effect of individual and/or sample and residual error (Figure 4, Table S6). Only a few of the identified mRGs showed truly uniform expression across samples (*Plekha7*, *Zfx*, *Ube2v1*, *Tspan15*) like the uRG *Oaz1*. Of the 27 novel mRGs optimized for the jejunum, we found eight cases of strain-dependent variation in expression (Table S6). None of the genes investigated dropped below the threshold *P*-value set for sex and strain-by-sex interaction.

We also determined the number of genes with less than two-fold variation of expression for each strain. This level of RG variation, alternatively expressed as variation of Δ*Cq* values in treated and untreated samples of ≤ ±0.5 at 100% amplification efficiency, has been used as cut-off for RG suitability [1]. A fluctuation less than two-fold is considered to stem largely from technical variance that similarly affects reference and target genes, for example due to unequal loading or differences in PCR efficiency across samples. It should not be confused with the final outcome of a RT-qPCR experiment, *i.e.* an n-fold change in target gene expression requiring accurate (multigene) normalization and calibration of a sufficiently large number of experimental samples. When samples were analysed for an individual mouse strain separately, OF1, C57Bl/6N or CD1, the cut-off of two-fold expression variation or less was met by 17, 24 and 25 genes, respectively (Table 2).

**Table 2:** Maximum range and arithmetic mean ± standard error of fold expression change for RG candidates in the mouse strains monitored.

Gene	OF1 (n = 7 or 9)	C57BL6/N (n = 4 to 6)	CD1 (n = 3)
*Plekha7*	**1.37** (1.17±0.13)	**1.58** (1.18±0.22)	**1.24** (1.12±0.10)
*Oaz1* 1	**1.58** (1.19±0.17)	**1.46** (1.17±0.14)	**1.20** (1.05±0.08)
*Zfx*	**1.56** (1.16±0.19)	**1.47** (1.20±0.16)	**1.33** (1.13±0.18)
*Tom1*	**1.74** (1.25±0.24)	**1.57** (1.27±0.23)	**1.16** (1.07±0.08)
*Hprt* 2	**1.76** (1.37±0.27)	**1.61** (1.26±0.24)	**1.73** (1.28±0.27)
*Tmem14c*	3.31 (1.53±0.61)	**1.58** (1.15±0.18)	**1.63** (1.19±0.26)
*B1 element* 3	**1.46** (1.16±0.15)	**1.57** (1.19±0.17)	**1.43** (1.17±0.18)
*Hjurp*	**1.70** (1.28±0.23)	**1.82** (1.30±0.34)	**1.47** (1.27±0.22)
*Cxx1b*	2.11 (1.41±0.41)	**1.99** (1.39±0.37)	**1.90** (1.42±0.45)
*D15Ertd30e*	**1.66** (1.35±0.24)	**1.67** (1.28±0.24)	**1.40** (1.18±0.20)
*Tspan15*	**1.82** (1.28±0.27)	**1.49** (1.16±0.16)	**1.23** (1.10±0.10)
*Ube2v1*	**1.64** (1.23±0.20)	**1.73** (1.27±0.25)	**1.19** (1.07±0.09)
*Zfyve19*	2.03 (1.36±0.25)	2.05 (1.38±0.37)	**1.38** (1.16±0.15)
*AI314976*	5.57 (2.69±1.75)	**1.54** (1.18±0.20)	**1.28** (1.08±0.12)
*B2 element* 3	**1.68** (1.23±0.19)	**1.70** (1.21±0.22)	**1.28** (1.12±0.11)
*Hadhb*	12.15 (2.05±2.92)	**1.31** (1.12±0.11)	**1.90** (1.28±0.35)
*Rps29* 1	**1.90** (1.26±0.23)	**2.00** (1.35±0.32)	**1.62** (1.23±0.24)
*Zfp598*	**1.64** (1.28±0.20)	**1.44** (1.17±0.15)	**1.35** (1.17±0.14)
*Atp6v0d1*	**1.74** (1.31±0.25)	**1.66** (1.19±0.22)	**1.28** (1.12±0.12)
Pcdha@	2.78 (1.65±0.72)	**1.81** (1.42±0.35)	**1.60** (1.21±0.34)
*Fbln1*	**1.80** (1.35±0.27)	**1.46** (1.17±0.16)	3.10 (1.50±0.81)
*Gsr*	6.60 (2.38±1.34)	2.44 (1.74±0.53)	**1.95** (1.47±0.47)
*Aldoa*	**1.69** (1.31±0.24)	2.32 (1.58±0.53)	2.61 (1.70±0.83)
*Rpl4* 1	2.02 (1.53±0.36)	**1.40** (1.17±0.17)	**1.63** (1.33±0.30)
Gag@	3.67 (2.11±0.93)	**1.80** (1.31±0.31)	**1.45** (1.18±0.22)
*Slc52a3*	5.86 (2.39±1.36)	5.12 (1.94±1.29)	3.49 (1.71±0.98)
*B3gnt3*	4.28 (2.77±0.96)	5.40 (2.39±1.76)	3.47 (1.68±1.01)
*Usmg2*	4.76 (2.10±1.28)	**1.50** (1.24±0.20)	**1.14** (1.06±0.07)
*St6galnac1*	19.92 (7.61±6.99)	3.09 (1.61±0.76)	2.37 (1.68±0.69)
*Fcer2a*	70.26 (32.18±27.64)	356.4 (135.9±148.8)	23.75 (8.69±13.04)

gene list was ordered according to Fig. 2.

values in bold: ≤ two-fold expression variation (minimum to maximum).

@: gene cluster.

1 uRG,

2 tRG,

3 rRG.

### Co-expression Analysis

**Figure 5:**
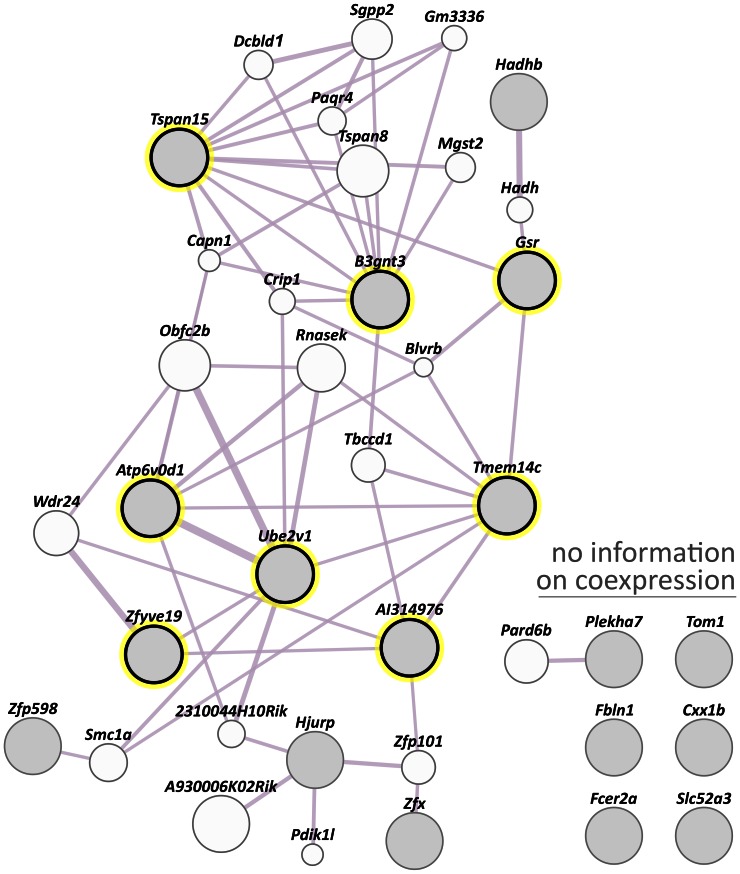
GeneMANIA co-expression analysis for the novel mRGs. Direct co-expression of novel mRGs (filled grey circles) is highlighted by a yellow surrounding circle. For the genes on the top right there was no information on co-expression. Two genes are linked if their expression levels are similar across conditions in a gene expression study, mostly derived from peer-reviewed publication data publicly accessible via GEO. A thicker line indicates a higher combined weight. The circle size depicts the extent of relatedness of a particular gene to the query genes. The cluster of Gag genes was not queried. Note that *D15Ertd30e* (NCBI gene identity number: 52238) and Pcdha@ (192162) were not listed in the GeneMANIA system.

Co-expression analysis across all tissues was performed using GeneMANIA containing data collected mostly from the Gene Expression Omnibus (GEO) and that are associated with a publication. No obvious overall co-expression was detected within the set of novel mRGs (Figure 5). An elevated weight score was determined only for one pair of direct coexpressors (0.21; *Atp6v0d1* and *Ube2v1*). The weight scores of seven other gene pairs were at least one order of magnitude lower (0.02 to 0.007; *Tmem14c* and *AI314976*, *Tmem14c* and *Gsr*, *Tspan15* and *B3gnt3*, *Zfyve19* and *Ube2v1*, *Tmem14c* and *Ube2v1*, *Zfyve19* and *AI314976*, *Atp6v0d1* and *Tmem14c*).

**Figure 6:**
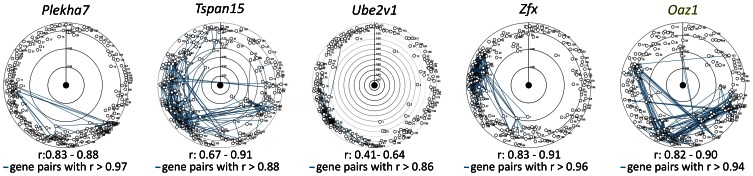
mRGs for which no significant fixed effect (strain, sex, strain by sex) was revealed are not closely correlated in the small intestine. Pearson product-moment correlation coefficients (*r*) were calculated for the 200 most correlated genes (circles) using the Co-Expression tool of Genevestigator (n = 3 microarray data sets). The co-expression networks constructed for the novel mRGs (gene symbols in black) and the uRG *Oaz1* (green) contained no case of coexpressed genes among the five genes analysed. Rank numbers are inversely proportional to the *r*-value.

In the target tissue, the co-expression of the novel mRGs was analysed with the ‘Co-Expression tool’ of Genevestigator. No coexpressed gene pairs were found when the networks of the novel mRGs that showed no significant fixed effect (*Tspan15*, *Ube2v1*, *Plekha7* and *Zfx*; see above) was analysed (Figure 6). For these four genes public SNP databases did not contain any SNP in the binding sites of the qPCR primers listed in Table 3. It is thus unlikely that this type of a genetic variation influences the expression data obtained with our RT-qPCR primers, at least for common mouse strains. Note that copy number variations of genomic regions were not addressed in this study.

**Table 3:** Sequences of oligonucleotide primers used in RT-qPCR.

Genesymbol	Accessionnumber	Forward primer (5′–3′)	Reverse primer (5′–3′)	Amplicon(bp)	Efficiency	Error^1^	Assay ID
							
*AI314976*	NM_207219	GGCGTCACTGACCTCTCCAT	TTGACTCAAACACCTCTTCGAGAA	97	77	13.4	8597^3^
*Aldoa*	NM_001177307	GTGTTGTGGGCATTAAGGTAGATAAG	CATCCAGCCCCTGGGTAGTT	81	95	2.9	8598^3^
*Atp6v0d1*	NM_013477	ATTTCAACGTGGACAATGGCTACTT	TCTCGCACTGCACCAGGTT	99	88	2	8599^3^
*B1 element*	multiple	TGGCGCACGCCTTTAATC	GCTGGCCTCGAACTCAGAAAT	46–73	56	1.4	Unpublished^4^
*B2 element*	multiple	CAATTCCCAGCAACCACATG	ACACCAGAAGAGGGCATCAGA	66–68	82	1.4	Unpublished^4^
*B3gnt3*	NM_028189	CCCCTACGAGATCCTCCTCC	CACGGATAGATTAGCTCGGCA	181	85	2.7	31982625a1^2^
*Fbln1*	NM_010180	ACCAGGCCGACATCATCTTC	TTCAGGACAGCGTAAAACGGG	142	68	1.8	6753822a2^2^
Gag@	multiple	TGGAGTAGGCTGGCCACCTA	CAGCCAAGCCAAACGCTATT	131	79	5.3	8609^3^
*Gm14743*	NM_001126321	GACGAGTGGTCCCCTGGAG	TGCTTATACCCAGTGACTGTGGTC	129	87	4	8611^3^
*Gsr*	NM_010344	TGACAACATCCCTACTGTGGTCTT	GGGTAAAGGCAGTCGAGTAGATTTTC	122	88	0.4	8604^3^
*Hadhb*	NM_145558	GCTAGAGCTGCACTTTCGGG	GCCACATTGCTTGTTTTCACTTC	110	85	2.2	21704100a2^2^
*Hjurp*	NM_198652	CGGTGCGGATACGAGCTT	CTCTGCGTTCTTAAAATACTCTTCATCT	187	84	0.8	8600^3^
*Hprt*	NM_013556	AGCTACTGTAATGATCAGTCAACG	AGAGGTCCTTTTCACCAGCA	198	82	0.5	3583^3^
*Oaz1*	NM_008753	GTGGTGGCCTCTACATCGAG	AGCAGATGAAAACGTGGTCAG	120	82	1.3	7110677a3^2^
Pcdha@	multiple	TGGAGGAGGCTGGCATTCTA	GGGTTGCCTGGTCCGTATT	163	84	8.5	8608^3^
*Plekha7*	NM_172743	GAACGCTTCCGTGCTCAAAG	GATCGACAAACAAGGCATCCT	103	86	0.1	27370088a2^2^
*Rpl4*	NM_024212	CCGTCCCCTCATATCGGTGTA	GCATAGGGCTGTCTGTTGTTTTT	147	84	0.4	30794450a1^2^
*Rps29*	NM_009093	CAACCGCCACGGTCTGAT	CATTCAAGGTCGCTTAGTCCAACT	110	86	1.2	8602^3^
*Slc52a3*	multiple^6^	ACCAGGGACCACCTTGAACACTA	CAGCAGCACGTCAGGAAAGAC	159	76	2.3	8610^3^
*St6galnac1*	NM_011371	GCCATCCACCATGACGAGAT	GCACTTGCGTCATTCTTCCA	159	91	4	6755510a1^2^
*Tmem14c*	NM_025387	CTGTCTCAGGATCCCAGGAATGT	CAAACTGGCTCCTGCGATTAA	129	83	4.7	8603^3^
*Tspan15*	NM_197996	CGGCAGAAATACAAAACCCTGG	CAGAAGGCACAGGTTGTCC	132	95	0.6	13278031a2^2^
*Zfp598*	NM_183149	GGTGCTCTACCAAGATGCGG	GGATGGGGATCAGGGCAAA	117	87	0.6	34147169a1^2^
*Zfyve19*	multiple^5^	GCTGTCAAGTTCACCCTCTTCAA	GGCTCGGCCACAATTCTTACA	57	90	5.4	8605^3^
*Ube2v1*	NM_023230	TGATAATTGGACCTCCACGAACAA	TCTCGGATCCACCACTCCAT	158	95	1.6	8606^3^
*Usmg2*	AJ290944	TTAGGGATGCAGAACACGTGAAA	CAACATCAACTCAAGCTAACATAGGAA	59	99	2.3	8607^3^

@: gene cluster.

*Pcdha*@: Pcdha cluster of genes binding to *Pcdha1, Pcdha10, Pcdha11, Pcdha12, Pcdha2, Pcdha3, Pcdha4, Pcdha5, Pcdha6, Pcdha7, Pcdha8, Pcdha9, Pcdhac1* and *Pcdhac2*.

1 mean squared error of the single data points fit to the regression line given in %.

assay details are accessible in the original publication or in the repositories for primer sequences (^2^PrimerBank or ^3^RTPrimerDB).

4 primer sequences provided by Vandesompele, J.

5 accession numbers: NM_001164827 and NM_028054.

6 accession numbers: NM_001164819 and NM_027172.

### Potential of Novel mRGs for Improvement of RT-qPCR Data

The value of the novel mRGs for improving multi-gene normalization of RT-qPCR data was demonstrated by ranking their expression stability in the experimental samples of inbred and outbred strains using the algorithm RefFinder. Single genes and sets of novel and former RGs as well as their combinations were analysed. As expected from their highly stable expression across the inbred and outbred samples analysed (Figure 2), the set consisting of the novel gene *Plekha7* and the uRG *Oaz1* showed superior expression stability (Table S7). Thus a better normalization can be achieved with fewer genes including novel mRGs.

Alternatively, the potential of a candidate set of RGs can be estimated by the standard error of the mean over different conditions, *i.e.*, the lower the standard error the better the set. This standard error is approximately equal to the square root of the mean variance of the RGs over the different conditions divided by their number. Applying this calculation to sets of representatives of the tRG, rRG and uRG classes (*Oaz1*, *Hprt*, *B1 element*, *B2 element*, *Rps29* and *Rpl4* (cumulative variance of 0.145), a reduced standard error was found for the set consisting of the top three novel mRGs (*Plekha7*, *Zfx* and *Ube2v1*; 0.140) and also for the top ranking RG pair (*Plekha7* and *Oaz1*; 0.129). We note that with the same or even a lower number of mRGs the same or a lower standard error can be achieved, which improves accuracy and reduces sample amount and costs.

## Discussion

For normalization of RT-qPCR-based expression analysis in a single tissue only some tRGs [14] and a few uRGs have been used because of their relatively stable expression across a multitude of cell types and tissues under varying experimental conditions [20], [24]. For the biological context of a single tissue, a context-specific meta-analysis might increase the number, uniformity in expression and functional diversity of putative RGs for normalization of RT-qPCR data.

In the past, mRGs have specifically been selected for pathological states including pathogenic infection and cancer, a tissue subset, developmental status, cell differentiation *etc.*, using mining of RNA expression data using ESTs [30], microarrays [39], [40], or a combination of sequencing- and hybridization based platforms to overcome the limitations of each approach [20] (Table S1). We now describe an approach for the substantial reduction of cross-platform microarray data required to select optimized mRGs for the context tissue, the jejunal section of the mouse small intestine. The microarray expression data mined was exclusively derived from this particular biological context. Our novel data mining protocol resulted in the identification of the novel mRGs *Plekha7*, *Zfx*, *Ube2v1* and *Tom1*. In the inbred and outbred samples analysed their expression varied two-fold or less in relation to the sample with the minimum steady-state RNA level (Figure 2). Hence the genes met the criterion for RG suitability of Δ*Cq* ≤ ±0.5 around average sample expression [1]. Similarly, we could show that the variation of two representatives of the uRG and tRG classes, *Oaz1* and *Hprt,* respectively, also remained below this threshold of expression stability (Figure 2). The combination of a novel mRG with an uRG, *Plekha7* and *Oaz1*, showed superior ranking in expression stability analysis with the tool RefFinder and the lowest cumulative variance. The fact that the novel top ranking mRGs had not been previously discovered, their highly stable expression, their assignments to diverse biological processes and the absence of pairwise co-expression among the genes demonstrate the success of our selection approach. The high level of expression uniformity of our novel mRGs can be beneficial for studies addressing the effects of small changes in expression about which little is known at present, largely due to the technical limitations. For example, circadian influences, diet changes or the length of time an organism is exposed to light, are thought to cause only minor changes in gene expression [41], [42], [43], [44]. Technical improvement such as the adoption of a tissue-specific normalization strategy in combination with advances in digital PCR [45] are expected to shed light on this yet neglected field soon.

The information on outliers with higher (*AI314976*) and lower levels of expression (*Tmem14c*, *Hadhb* and *Gsr*; Figure 2) may help to broaden our understanding of the regulation of these genes. For example the deviant expression could be the result of pathogenic infection or of allelic or physiological variants with aberrant transcript regulation.

RNA-seq, a massively parallel sequencing method for transcriptome analysis, will improve the understanding of the repertoire and regulation of transcript isoforms [46]. *E.g*., for the mRG *Plekha7*, the gene top ranked by RT-qPCR (Figure 2), five transcript isoforms are predicted in the UniProt database. Since no information on the tissue-specific expression or function of these isoforms was available, we randomly chose a single exon contained in all predicted isoforms as target for our RT-qPCR primers. Like exon microarrays [47], RNA-seq would also allow to identify circadian alternative splicing. The ability to identify genes that are affected by (peripheral) circadian clock mechanisms, but that can also be transcribed into isoforms not subjected to this type of regulation, could further increase the repertoire of transcripts for context-specific selection of mRGs.

Another way to improve context-specific selection of mRGs in the mouse would be to use a broader range of allelic variants. As an example, the Collaborative Cross (CC), a large set of recombinant inbred lines derived from genetically diverse progenitors [48], provides access to a more complete catalogue of variation [49].

In conclusion, we have performed data mining across microarray platforms to identify mRGs. This was followed by RT-qPCR-based determination of expression uniformity, where representative genes of the tRG, uRG and rRG classes were included for comparison. This work shows that, with a meta-analysis of RNA expression data restricted to a particular context tissue, mRGs of high expression stability can be identified. The novel mRGs optimized for the mouse jejunum should complement or replace genes from former RG classes such as tRGs (Table S8) for RT-qPCR-based expression monitoring in this tissue. When the set of invariant mRGs specifically selected for a specific tissue is used for normalization of microarray expression data of coding and small non-coding RNAs in this context [17], [31], [50], differentially expressed transcripts and key pathways can be identified with higher sensitivity [31]. Irrespective of whether transcriptome data are obtained by microarray or sequencing-based technologies, the strategy of context tissue-specific selection of RGs should be considered for expression studies of other target tissues. The community tool RefGenes of the Genevestigator database developed for the identification of reliable and condition-specific candidate RGs for the normalization of RT-qPCR data could be helpful in this regard by allowing the mining of expression data of at least a single microarray platform [32]. In addition, Automated Microarray Data Analysis (AMDA) has been developed for the analysis of microarray expression data from multiple platforms [51]. Its next release could easily implement a tool for the selection of mRGs (Dimos Kapetis, personal communication).

## Materials and Methods

### Ethics Statement and Animal Welfare

This study was carried out in accordance with the recommendations of the Austrian national law (TVG 1988, BGBl. Nr. 501/1989) and of the European Parliament and of the Council on the protection of animals used for scientific purposes (Directive 2010/63/EU). Jejunal samples of OF1 mice used for microarray analysis were derived from a previous nutritional study conducted at the Institute of Nutrition, University of Veterinary Medicine Vienna and approved by the ethics committee of the University and the national ethics committee for animal experiments (GZ: 68.205/0042– BrGT/2006). No animal trial was conducted on the C57Bl/6N and CD1 mice according to the above stated laws (§2 and paragraph 3 of article 1 in chapter 1). Animals were euthanized by vertebral dislocation according to the Directive 2010/63/EU (article 6 of chapter 1 and annex IV). One author of this work (CB) is a trained veterinarian and was responsible for animal welfare. The health status of the mice was checked daily.

### Mouse Strains

For the internal DNA microarray expression data seven weeks old male mice of the vigorous and productive outbred strain OF1 [52] were purchased from the Research Institute for Laboratory Animal Breeding (Himberg, Austria). The OF1 strain at this breeding institution descended from a breeding colony obtained by Iffa Credo (L’Arbresle, France; now Charles River Laboratories International, Wilmington, USA) and passed genetic re-management started in February 2003. The internal expression data set addressed the effect of a custom maize diet (Ssniff, Soest, Germany) fed *ad libitum* to mice kept under conventional housing conditions.

C57Bl/6N inbred and CD1 outbred mice (Biomodels Austria, Vienna, Austria) were used together with OF1 mice for expression analysis by RT-qPCR.

Until sampling mice were kept at the Institute of Laboratory Animal Science (University of Veterinary Medicine Vienna) in macrolon cages of varying sizes under a 12 hours light/dark cycle. Housing was in accordance with the standards given by the national ethics committee for animal experiments.

### Intestinal Tissue Sampling

Before sampling of the jejunum a starvation period of three hours during the light cycle was introduced. During this time food, but not water was withheld from the mice. Mice were sacrificed by vertebral dislocation. The small intestine beginning from the pylorus to the ileocaecal junction was dissected immediately and divided into two fragments of similar length. A 2 cm segment of the jejunum covering the 1 cm proximal and 1 cm distal regions around the angular point was dissected. Tissue samples were shock frozen in 2-methyl-butane (Merck, Darmstadt, Germany) cooled with liquid nitrogen. After shock freezing the samples were transferred to cryo tubes (Bertoni, Vienna, Austria) and stored in liquid nitrogen until further processing.

The daytime of sampling was not recorded nor randomized between the experimental groups. This does not allow exclude bias from (peripheral) circadian gene regulation [47], [53]. Hence, the value of the internal microarray data set for the context originally addressed (alimentation) is reduced. However, the data set is valuable when the selection of RGs is addressed considering that the common RG concept is incompatible with regulation by the circadian clock.

### RNA Isolation from Murine Jejunum

50 mg intestinal tissue was homogenized in 700 µL of a monophasic solution of phenol and guanidinium isothiocyanate (Qiazol Lysis Reagent, Qiagen, Hilden, Germany) using 2 ml tubes pre-filled with 1.4 mm diameter ceramic spheres (MagNA Lyser Green Beats, Roche Diagnostics, Mannheim, Germany). Tissues were homogenized twice for 20 s in the MagNA Lyser instrument (Roche Diagnostics) at 6,500 rpm. The lysate was stored at −80°C until further processing. After the addition of 1/5 (v/v) chloroform and centrifugation at 4°C for 15 min at 12,000×g, the aqueous phase was transferred to a new tube. The subsequent processing of the extraction of cellular RNA including small RNAs was performed with the miRNeasy Kit (Qiagen) in combination with the QIAcube (Qiagen) for automated sample preparation.

RNA concentration was determined by spectrophotometry using the Hellma TrayCell (Hellma, Müllheim, Germany) in combination with the BioPhotometer (Eppendorf, Hamburg, Germany). RNA integrity was assessed with the RNA 6000 Nano Chip kit (Agilent Technologies, Santa Clara, USA) on the 2100 Bioanalyzer (Agilent Technologies). RNA Integrity Number (RIN) values were calculated by the 2100 Expert software (version B.02.03.SI307; Agilent Technologies). RIN values of >8 (inter-sample variation of ΔRIN ≤0.5) and >7 were used as threshold for RNA integrity in microarray and RT-qPCR analyses, respectively.

To remove contaminating DNA in RNA samples subjected to RT-qPCR, 16 µl of RNA (125 µg/µL) was mixed with 2 µL of 10×Reaction Buffer and 2 µL RQ1 RNase-Free DNase (1 U/µL; Promega, Leiden, Germany) and incubated at 37°C for 30 min. The reaction was terminated by adding 2 µL of 20 mM EGTA (pH 8) and incubating at 65°C for 10 min. For spin-column purification the E.Z.N.A.® MicroElute® Total RNA Kit (Omega Bio-tek, Norcross, USA) was used.

### Internal Expression Data Set Obtained with Applied Biosystems Mouse Genome Survey Microarray

DIG-labeled cDNA probes were reverse-transcribed from 40 µg total RNA using the Chemiluminescent RT-Labelling kit (Applied Biosystems) as described by the manufacturer. Array hybridization, chemiluminescence detection, image acquisition and analysis were performed using the Chemiluminescence Detection Kit (Applied Biosystems) and the 1700 Chemiluminescence Microarray Analyzer (Applied Biosystems) following the instructions of the manufacturer. Each microarray was first pre-hybridized at 55°C for 1 h in hybridization buffer with blocking reagent. Oligo-dT primed, DIG-labeled cDNA targets were fragmented, mixed with the internal control target and then hybridized to the equilibrated microarrays in a volume of 1.5 ml at 55°C for 16 h. After hybridization, the arrays were washed with hybridization wash buffer and chemiluminescence rinse buffer. The enhanced chemiluminescent signals were generated by incubating arrays with an alkaline phosphatase conjugated anti-digoxigenin antibody followed by incubation with the chemiluminescence enhancing solution and final addition of the chemiluminescence substrate. Four images were collected for each microarray using the ABI 1700 Chemiluminescent Microarray Analyzer. The images were auto-gridded and the chemiluminescence signals were quantified, corrected for background and spot and spatially normalized.

Microarray data are accessible at the Gene Expression Omnibus under the accession GSE44396.

### Meta-Analysis of Microarray Expression Data for the Mouse Intestine

The key words “*Mus musculus*” and either “jejunum”, “duodenum“, “ileum” or “small intestine” were queried against the public databases Gene Expression Omnibus [54] and Array Express [22] using default search settings. A total of 220 intestinal microarrays comprising 23 arrays for the duodenum, 53 for the jejunum, 63 for the ileum and 81 arrays from unassigned small intestinal samples were downloaded (Table S2). Collectively, the internal and external microarray data sets crossed were derived from nine platforms. Data lacking information on pre-normalization, cell culture experiments and probes without NCBI gene symbol were excluded. As a detection *P*-value was not available for all data sets, quality-weighting [55] was not performed. For several data sets raw data were unavailable (Table S2) precluding the use of the parametric framework suggested by [56].

Raw data or if unavailable data scaled with the Affymetrix MAS5 normalization algorithm were quantile normalized using the GenStat software (11th edition for Windows; VSN International Ltd, Hemel Hempstead, United Kingdom) using the option of the arithmetic mean according to the recommendation of the software provider (Baird D, Analysis of Microarray Data, 2011). Quantile normalized data was log_2_ transformed. After normalization spots lacking a gene symbol were removed, genes detected in less than half of the available microarrays were omitted, and genes detected by more than one set of probes were averaged. Subsequently, the data of each array were transformed to zero mean and unit variance, *i.e.* studentized [57]. The resulting data were subjected to two meta-analysis protocols. To avoid computer memory problems during subsequent ANOVA runs, a significant reduction of data was achieved as follows. The CV% was calculated for each gene across all arrays and only the 10% genes with the lowest CV% were processed further.

In the first approach, the 10% genes with the lowest CV% were submitted to a one-way ANOVA with microarray experiment as fixed effect using PROC MIXED of the SAS 9.1 software (SAS Institute, Cary, USA). Genes were ranked according to their *P-*values determined by the Wald χ^2^ test. Obviously, differentially expressed genes should primarily have low *P*-values, while candidate mRGs should primarily have high *P*-values. Thus ranking the genes according to *P*-value should lead to the best candidate mRGs among those with high *P*-values.

With the second strategy, we aimed for the same overall goal of identifying candidate mRGs according to their uniformity of expression among all genes. An ANOVA was performed independently on the independent experiments using the log_2_ transformed data. The variances were weighted to compensate for differences in experimental design among studies using the formula 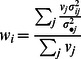, where *i* is the gene, *j* the microarray platform, *ν* the degrees of freedom given by the experimental design and *σ_ij_^2^* is the variance of the *i* ^th^ gene, in the *j* ^th^ microarray platform. The ANOVA was run using the “lm” command of the statistical program package “R” (www.r-project.org). Finally, the genes were ranked based on the “weighted” *P*-value to choose the most uniformly expressed genes.

Note that in both protocols of meta-analysis, the impact of the different biological conditions studied in the different experiments was not accounted for.

The preferred reporting items for systematic reviews and meta-analyses (PRISMA) checklist is presented as Checklist S1.

### RT-qPCR

RNA with a RIN value of at least 7 (sample range: 7.8 to 9.6) was reverse-transcribed using the High-Capacity cDNA Reverse Transcription Kit (Applied Biosystems, Foster City, USA) according to the manufacturer’s instructions. The cDNA synthesis reaction consisted of 2 µL 10×RT Buffer, 0.8 µL 25×dNTP Mix (100 mM of each dNTP), 2 µL 10×RT Random Primers, 50 U Multiscribe Reverse Transciptase, 10 µL of RNA (200 ng/µL) and 4.2 µL nuclease-free water. The reaction was incubated for 10 min at 25°C followed by 120 min at 37°C and terminated by heating at 85°C for 5 s. Duplicate experimental cDNAs and a single minus RT control of the pooled experimental RNAs were analysed. A fluorescent dye-based qPCR targeting the gene *Hprt* was performed to assess the outcome of RT (assay oligonucleotides in Table 3).

The 10 µl-qPCR included 1×reaction buffer BD (80 mM Tris-HCl, 20 mM (NH_4_)_2_SO_4_; Solis Biodyne, Tartu, Estonia), 3.5 mM MgCl_2_ (Solis Biodyne), 0.2 mM of each dNTP (Solis Biodyne) 0.4×EvaGreen (Biotium, Hayward, USA), 200 nM of each primer and 0.05 U/µL hot-start *Taq* DNA polymerase (HOT FIREPol® DNA polymerase; Solis Biodyne). The liquid handling system EpMotion 5075 TMX (Eppendorf) was used for the set-up of the 384-well microtiter plate. The qPCR was run on the LightCycler 480 (software version 1.5.0; Roche Diagnostics) using the cycling conditions of 1×95°C/15 min and 40×(95°C/15 s, 60°C/30 s and 72°C/20 s). The specificity of the amplicon was concluded from its melting curve.

The primer pairs were taken from public resources for PCR primers (PrimerBank [58], or RTPrimerDB [59]) if one of the primers targeted an exon/exon boundary or flanked an intronic sequence of >500 bp. If such primer pairs were not publicly available, they were designed with Primer Express 2.0 (Applied Biosystems). The annealing temperature range (58 to 62°C) and the amplicon size (<200 bp) were changed in the default settings of the program. Primers were tested for dimerization using the PerlPrimer software [60] or the primer analysis tool NetPrimer (Premier Biosoft International, Palo Alto, USA). The efficiency of amplification of a primer pair was determined using serial dilutions (1∶5, 1∶10, 1∶20 and 1∶30) of an equimolar mix of the experimental samples as the template. The *Cq* values were computed from the second derivative method of the LightCycler software. An error value (mean squared error of the single data points fit to the regression line) of <0.2 was considered as acceptable based on the LightCycler 480 manual. This threshold was only exceeded in the case of the lowly expressed gene *Ube2v1* (error: 0.241). Primer sequences and assay details are listed in Table 3.

When an assay had to be run on two 384-well microtiter plates, the range of expression variation was depicted by the difference between the minimum and the individual *Cq* values.

For *Cq* values generated at less than 100% amplification efficiency (E), the equation


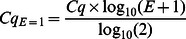
, where *Cq*_E_ is the uncorrected *Cq* value, was used to calculate a putative *Cq* at E = 100%. Note that amplification efficiency was determined by the formula 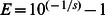, were s is the slope of the graph obtained by plotting the *Cq* versus the log_10_ of the cDNA input.

The RT-qPCR data comply with the MIQE guidelines [2].

### Evaluation of G-Spots in Affymetrix Probes Sets

Probe sets of genes exhibiting discordant expressions between Affymetrix microarrays and RT-qPCR were downloaded from NetAffx™ Analysis Center (www.affymetrix.com/analysis/index.affx) to identify sequences of four or more guanines termed G-spots.

### Sanger Sequencing

Sequence analysis of the amplicon being identical to the predicted protein coding genes Gm4268, Gm3817, Gm4569, and Gm2251 was performed at LGC Genomics (Berlin, Germany). The sequence chromatograms were evaluated with the CodonCode Aligner version 3.7.1 (CodonCode Corporation, Dedham, USA).

### Grubbs’ Test for Outliers

Outliers were detected at a significance level of 0.01 by the Grubbs’ outlier test [61], also known as the maximum normed residual test, using Microsoft excel 2010.

### Assessment of Expression Stability Irrespective of Strain, Sex, Strain by Sex Interaction, Random Effect of Individual and Genetic Homogeneity

Factor independent expression stability was assessed based on the *P* values resulting from a linear mixed models analysis of variance with mouse strain, sex and their interaction as fixed effects and the mouse individuals as random effect (PROC MIXED, SAS 9.1 software). The comparisons of the means of outbred versus inbred mouse strains were carried out by defining the respective contrasts in the SAS statements. *P*-values of >0.05 and >0.1 were assumed for the F test of fixed effects and for the χ^2^ statistic of the likelihood ratio test for the random effect, respectively.

### Co-expression Analyses

With GeneMANIA coexpressed genes were identified based on functional association data such as protein and genetic interactions, pathways, co-expression, co-localization, and protein domain similarity [62]. The default method, automatically selected weighting method, was used when combining all networks into the final composite. In case of our search list with more than five genes, GeneMANIA assigns weights to maximize connectivity between all input genes using the ‘assigned based on query gene’ strategy. According to this strategy the weights are chosen automatically using linear regression, to make genes on the search list interact as much as possible with each other and as little as possible with genes not on this list.

To exclude that the novel mRGs are highly correlated in the specific target tissue, their co-expression was analysed with the ‘Co-Expression tool’ of Genevestigator [19]. While expression data restricted to the jejunum were not available in this reference expression database for the meta-analysis of transcriptomes, microarray studies targeting the small intestine were used instead (Genevestigator numbers MM-00274 (GEO accession number GSE8065), MM-00289 (GSE8582) and MM-00314 (GSE9533)).

### Comparison of Expression Stability of Two Sets of Putative RGs using RefFinder Analysis

The web-based comprehensive tool RefFinder (http://www.leonxie.com/referencegene.php) was used to demonstrate the potential improvement of RT-qPCR normalization [12], [63], [64]. RefFinder integrates the computational programs geNorm [6], Normfinder [8], BestKeeper [9] and the comparative Δ*Cq* method [13]). The integration of four statistical programs helps to overcome the weaknesses of the individual statistical methods such as sensitivity to co-regulation in case of geNorm [8], [65], [66], [67] and the comparative Δ*Cq* method [64], not-accounting for systematic errors during sample preparation by the NormFinder approach [64] and a too strict inter-sample variance allowed by BestKeeper [68].

In addition, superiority of a set of non-correlated putative RGs for RT-qPCR normalization was concluded if the standard error of the mean over different conditions approximated by 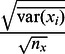 was lower for a set of genes in comparison to the one calculated for an alternative set. In the term the expression variance is given as *Var* and *n* represents the number of genes in the gene set.

When a target transcript was assayed on two 384-well microtiter plates, inter-assay variation was compensated. This correction was done by substracting the mean *Cq* of the second plate from the mean *Cq* of the first plate. This difference was added to the individual *Cq* values of the second plate.

## Supporting Information

Table S1 Genome-wide studies to identify mRGs for normalization of expression in different biological contexts.
(DOCX)

Table S2 Microarray experiments used for meta-analysis targeting the small intestine or its sections.
(DOCX)

Table S3 Microarray types contributed to the ranking of the top 100 RGs of jejunum-restricted meta-analysis I.
(XLSX)

Table S4 Rank order lists of genes identified by meta-analyses restricted to the duodenum, the ileum or the small intestine.
(XLSX)

Table S5 Microarray types contributed to the ranking of the top 100 RGs of jejunum-restricted meta-analysis II.
(XLSX)

Table S6 mRGs stably expressed irrespective of strain, sex, strain by sex interaction and random effect.
(DOC)

Table S7 Expression stability analysis performed with RefFinder.
(XLSX)

Table S8 Details on the RT-qPCR normalization in arbitrarily selected studies of the mouse small intestine.
(DOCX)

Checklist S1 PRISMA 2009 Checklist.
(DOC)
