# Supplementary material for: Cross-Platform Microarray Meta-Analysis for the Mouse Jejunum Selects Novel Reference Genes with Highly Uniform Levels of Expression
Source: PLoS One. 2013 May 9;8(5):e63125. doi: 10.1371/journal.pone.0063125 (PMC3650031; doi:10.1371/journal.pone.0063125)
Supplement: Table S1 — Genome-wide studies to identify mRGs for normalization of expression in different biological contexts. (DOCX) [file pone.0063125.s001.docx]

**Table S1.** Genome-wide studies to identify mRGs for expression normalisation in different biological contexts.

| **Study** | **Organism** | **Biological context** | **RNA profiling platform** | **Microarray probe length (nts)** | **Array no.** | **Gene exclusion criteria** | **Ranking criterion** | **Platform no.** | **qPCR validation** |
| --- | --- | --- | --- | --- | --- | --- | --- | --- | --- |
| [[1](#_ENREF_1)] | *Homo sapiens* | melanoma and renal carcinoma cell lines | cDNA microarray | various | 384 | missing spots | SD and fluorescence intensity | 1 | yes |
| [[2](#_ENREF_2)] | *Arabidopsis thaliana* | Arabidopsis development | microarray | 25 | 721 | no exclusion | CV% | 1 | yes |
| [[3](#_ENREF_3)] | *H. sapiens* | heart pathologies | oligonucleotide microarray | not specified | 75 | no exclusion | two subsets statistically compared | 1 | yes |
| [[4](#_ENREF_4)] | *H. sapiens* | non-small lung cancer | microarray | 25 | 82 | no exclusion | integrative correlation and SD | 1 | yes |
| [[5](#_ENREF_5)] | *H. sapiens* | hepatitis C virus (HCV) induced hepatocellular carcinoma (HCC) | microarray | 25 | 72 | no exclusion | SD | 1 | yes |
| [[6](#_ENREF_6)] | *Canis lupus familiaris* | canine articular tissues | microarray | 60 | 26 | t test | fold change and CV% | 1 | yes |
| [[7](#_ENREF_7)] | *H. sapiens* | set of tissues | microarray | 25 | 281 | contrast analysis | fold change and regression | 1 | yes |
| [[8](#_ENREF_8)] | *H. sapiens/Mus musculus domesticus* | set of tissues | microarray | 25 | 13629/2543 | no exclusion | CV%, maximum fold-change and expression level | 1/1 | PCR |
| [[9](#_ENREF_9)] | *M. m. domesticus* | set of tissues | microarray | 25 | 1968 | no exclusion | CV% and SD | 1 | yes |
| [[10](#_ENREF_10)] | *H. sapiens* | multiple normal and cancer tissues | microarray, EST, SAGE | 25 | 77/326/567 | 0's proportion, mean expression and CV% | z test and clustering | 3 | yes |
| [[11](#_ENREF_11)] | *Oryza sativa* | organ, development, biotic and abiotic conditions | microarray | 25 | 331 | no exclusion | CV%, Euclidean distance | 1 | yes |
| [[12](#_ENREF_12)] | *Rattus norvegicus* | neuronal differentiation of pheochromocytoma cells | microarray | 50 | 12 | no exclusion | CV% | 1 | yes |
| [[13](#_ENREF_13)] | *H. sapiens* | physiological states | microarray | 25 | 4804 | no exclusion | intensity, presence, SD, fold change | 1 | no |
| [[14](#_ENREF_14)] | *Escherichia coli* | recombinant protein production | microarray | 25 | 240 | same transcription unit | CV% | 1 | yes |
| this work | *M. m. domesticus* | jejunal section of small intestine | microarray | various | 220 | CV% | *χ²* test | 9 | yes |

SD: standard deviation

**References**

1. Jin P, Zhao Y, Ngalame Y, Panelli MC, Nagorsen D, et al. (2004) Selection and validation of endogenous reference genes using a high throughput approach. BMC Genomics 5: 55.

2. Czechowski T, Stitt M, Altmann T, Udvardi MK, Scheible WR (2005) Genome-wide identification and testing of superior reference genes for transcript normalization in Arabidopsis. Plant Physiol 139: 5-17.

3. Shulzhenko N, Yambartsev A, Goncalves-Primo A, Gerbase-DeLima M, Morgun A (2005) Selection of control genes for quantitative RT-PCR based on microarray data. Biochem Biophys Res Commun 337: 306-312.

4. Saviozzi S, Cordero F, Lo Iacono M, Novello S, Scagliotti GV, et al. (2006) Selection of suitable reference genes for accurate normalization of gene expression profile studies in non-small cell lung cancer. BMC Cancer 6: 200.

5. Waxman S, Wurmbach E (2007) De-regulation of common housekeeping genes in hepatocellular carcinoma. BMC Genomics 8: 243.

6. Maccoux LJ, Clements DN, Salway F, Day PJ (2007) Identification of new reference genes for the normalisation of canine osteoarthritic joint tissue transcripts from microarray data. BMC Mol Biol 8: 62.

7. Lee S, Jo M, Lee J, Koh SS, Kim S (2007) Identification of novel universal housekeeping genes by statistical analysis of microarray data. J Biochem Mol Biol 40: 226-231.

8. de Jonge HJ, Fehrmann RS, de Bont ES, Hofstra RM, Gerbens F, et al. (2007) Evidence based selection of housekeeping genes. PLoS One 2: e898.

9. Frericks M, Esser C (2008) A toolbox of novel murine house-keeping genes identified by meta-analysis of large scale gene expression profiles. Biochim Biophys Acta 1779: 830-837.

10. Kwon MJ, Oh E, Lee S, Roh MR, Kim SE, et al. (2009) Identification of novel reference genes using multiplatform expression data and their validation for quantitative gene expression analysis. PLoS One 4: e6162.

11. Narsai R, Ivanova A, Ng S, Whelan J (2010) Defining reference genes in Oryza sativa using organ, development, biotic and abiotic transcriptome datasets. BMC plant biology 10: 56.

12. Zhou L, Lim QE, Wan G, Too HP (2010) Normalization with genes encoding ribosomal proteins but not GAPDH provides an accurate quantification of gene expressions in neuronal differentiation of PC12 cells. BMC Genomics 11: 75.

13. Cheng WC, Chang CW, Chen CR, Tsai ML, Shu WY, et al. (2011) Identification of reference genes across physiological states for qRT-PCR through microarray meta-analysis. PloS one 6: e17347.

14. Zhou K, Zhou L, Lim Q, Zou R, Stephanopoulos G, et al. (2011) Novel reference genes for quantifying transcriptional responses of Escherichia coli to protein overexpression by quantitative PCR. BMC molecular biology 12: 18.
