# Supplementary material for: Cross-Platform Microarray Meta-Analysis for the Mouse Jejunum Selects Novel Reference Genes with Highly Uniform Levels of Expression
Source: PLoS One. 2013 May 9;8(5):e63125. doi: 10.1371/journal.pone.0063125 (PMC3650031; doi:10.1371/journal.pone.0063125)
Supplement: Table S2 — Microarray experiments used for meta-analysis targeting the small intestine or its sections. (DOCX) [file pone.0063125.s002.docx]

**Table S2** Microarray experiments used for meta-analysis targeting the small intestine or its sections.

| **Study issue (reference)** | **Mouse strain** | **Array no.** | **Array type** | **Probe length (nt)** | **Label** | **Signal type** | **GEO (or Array Express) accession** | **Intestinal section studied** |
| --- | --- | --- | --- | --- | --- | --- | --- | --- |
| *Gata4* knock-out [[1](#_ENREF_1)] | C57BL/6, C57BL/6J x SJL/J | 9 | I | 25 | fluorophore (SAPE) | raw | GSE11194 | J, I |
| PUFA diet [[2](#_ENREF_2)] | C57BL/6J | 2 | I | 25 | fluorophore (SAPE) | MAS5 | GSE11936 | S |
| metagenome effects [[3](#_ENREF_3)] | NMRI | 18 | I | 25 | fluorophore (SAPE) | raw | GSE5198 | I |
| *Pten* knock-out [[4](#_ENREF_4)] | not specified | 5 | I | 25 | fluorophore (SAPE) | raw | GSE6078 | S |
| *Hfe* knock-out [[5](#_ENREF_5)] | C57BL/6, DBA/2 | 12 | I | 25 | fluorophore (SAPE) | MAS5 | GSE7357 | D |
| high fat/low fat diet [[6](#_ENREF_6)] | C57BL/6J | 18 | I | 25 | fluorophore (SAPE) | MAS5 | GSE8582 | S |
| immunological challenge [[7](#_ENREF_7)] | C57Bl/6J | 9 | I | 25 | fluorophore (SAPE) | dChip signal | GSE9018 | S |
| *Plagl2* knock-out [[8](#_ENREF_8)] | Swiss Webster x 129/SvJ | 8 | I | 25 | fluorophore (SAPE) | dChip signal | GSE9123 | S |
| RNA profiling across tissues | not specified | 23 | II | cDNA | fluorophores Cy3 and Cy5 | raw | GSE1701 | S |
| RNA profiling across tissues [[9](#_ENREF_9)] | C57BL/6J | 2 | II | cDNA | fluorophores Cy3 and Cy5 | raw | GSE2168 | D |
| RNA profiling across tissues [[10](#_ENREF_10)] | C57BL/6J | 12 | II | cDNA | fluorophores Cy3 and Cy5 | raw | GSE2178 | D, J, I |
| RNA profiling across tissues [[11](#_ENREF_11)] | not specified | 2 | IIIa, b, c | cDNA | fluorophores Cy3 and Cy5 | raw | GSE3098 | S |
| high fat diet [[12](#_ENREF_12)] | C57BL/6J, A/J | 4 | IV | 25 | fluorophore (SAPE) | raw | GSE3433 | S |
| *Por* knock-out [[13](#_ENREF_13)] | C57BL/6 | 24 | IV | 25 | fluorophore (SAPE) | MAS5 | GSE4257, GSE4258 | J |
| *Klf9* knock-out [[1](#_ENREF_14)4] | C57BL/6J | 10 | IV | 25 | fluorophore (SAPE) | raw | GSE6443 | S |
| RNA profiling [[1](#_ENREF_15)5] | BALB/c | 10 | V | cDNA | colorimetric^1^ | median signal | GSE4715 | I |
| nutritional diet study [16] | C3H | 24 | VI | 25 | fluorophore (SAPE) | MAS5 | GSE1392 | J, I |
| immunological challenge [[1](#_ENREF_16)7] | C57BL/6J | 20 | VI | 25 | fluorophore (SAPE) | MAS5 | GSE7013 | D, J, I |
| RNA profiling [[1](#_ENREF_17)8] | CD1 | 27 | VI | 25 | fluorophore (SAPE) | MAS5 | GSE849 | D |
| tumour study [[1](#_ENREF_18)9] | Apc1638N | 3 | VII | 25 | fluorophore (SAPE) | MAS5 | GSE13298 | D, J, I |
| RNA profiling across tissues [20] | C57BL/6 | 4 | VIII | 25 | fluorophore (SAPE) | dChip signal | (HGMP2) | J |
| alimentation study (this work) | OF1 | 9 | IX | 60 | chemolumi-nescence^2^ | raw | to be assigned | J |

Underline styles: single line (mixed genetic background), two lines (outbred strain)

Microarray platform: I, Mouse Genome 430A 2.0 Array (Affymetrix); II, IncyteMouseGEM1 (non commercial); IIIa, 20K Riken cDNA array; IIIb, Spotted Riken cDNA array 20-40K; IIIc, Spotted Riken cDNA array 40-60K; IV, Mouse Genome 430A Array (Affymetrix); V, NTU_CGM_MCF Mouse 6.1k Microarray (non-commercial); VI, MG-U74A, B, C (Affymetrix); VII, Mouse Genome 430 2.0 Array (Affymetrix); VIII, MG-U74Av2 (Affymetrix); IX, Gene Expression Array System (Applied Biosystems)

intestinal section studied: D, duodenum; J, jejunum; I, ileum; S, small intestine

SAPE: Streptavidin Phycoerythrin

^1^details of this colorimetric method were not specified

^2^chemoluminescence generated by alkaline phosphatase mediated substrate hydrolysis

**References**

1. Battle MA, Bondow BJ, Iverson MA, Adams SJ, Jandacek RJ, et al. (2008) GATA4 is essential for jejunal function in mice. Gastroenterology 135: 1676-1686 e1671.

2. van Schothorst EM, Flachs P, Franssen-van Hal NL, Kuda O, Bunschoten A, et al. (2009) Induction of lipid oxidation by polyunsaturated fatty acids of marine origin in small intestine of mice fed a high-fat diet. BMC Genomics 10: 110.

3. Rawls JF, Mahowald MA, Ley RE, Gordon JI (2006) Reciprocal gut microbiota transplants from zebrafish and mice to germ-free recipients reveal host habitat selection. Cell 127: 423-433.

4. He XC, Yin T, Grindley JC, Tian Q, Sato T, et al. (2007) PTEN-deficient intestinal stem cells initiate intestinal polyposis. Nat Genet 39: 189-198.

5. Coppin H, Darnaud V, Kautz L, Meynard D, Aubry M, et al. (2007) Gene expression profiling of Hfe-/- liver and duodenum in mouse strains with differing susceptibilities to iron loading: identification of transcriptional regulatory targets of Hfe and potential hemochromatosis modifiers. Genome Biol 8: R221.

6. de Wit NJ, Bosch-Vermeulen H, de Groot PJ, Hooiveld GJ, Bromhaar MM, et al. (2008) The role of the small intestine in the development of dietary fat-induced obesity and insulin resistance in C57BL/6J mice. BMC Med Genomics 1: 14.

7. Peterson DA, McNulty NP, Guruge JL, Gordon JI (2007) IgA response to symbiotic bacteria as a mediator of gut homeostasis. Cell Host Microbe 2: 328-339.

8. Van Dyck F, Braem CV, Chen Z, Declercq J, Deckers R, et al. (2007) Loss of the PlagL2 transcription factor affects lacteal uptake of chylomicrons. Cell Metab 6: 406-413.

9. Hutton JJ, Jegga AG, Kong S, Gupta A, Ebert C, et al. (2004) Microarray and comparative genomics-based identification of genes and gene regulatory regions of the mouse immune system. BMC Genomics 5.

10. Zhang J, Moseley A, Jegga AG, Gupta A, Witte DP, et al. (2004) Neural system-enriched gene expression: relationship to biological pathways and neurological diseases. Physiol Genomics 18: 167-183.

11. Ravasi T, Suzuki H, Pang KC, Katayama S, Furuno M, et al. (2006) Experimental validation of the regulated expression of large numbers of non-coding RNAs from the mouse genome. Genome Res 16: 11-19.

12. Kondo H, Minegishi Y, Komine Y, Mori T, Matsumoto I, et al. (2006) Differential regulation of intestinal lipid metabolism-related genes in obesity-resistant A/J vs. obesity-prone C57BL/6J mice. Am J Physiol Endocrinol Metab 291: E1092-1099.

13. Mutch DM, Crespy V, Clough J, Henderson CJ, Lariani S, et al. (2006) Hepatic cytochrome P-450 reductase-null mice show reduced transcriptional response to quercetin and reveal physiological homeostasis between jejunum and liver. Am J Physiol Gastrointest Liver Physiol 291: G63-72.

14. Simmen FA, Xiao R, Velarde MC, Nicholson RD, Bowman MT, et al. (2007) Dysregulation of intestinal crypt cell proliferation and villus cell migration in mice lacking Kruppel-like factor 9. Am J Physiol Gastrointest Liver Physiol 292: G1757-1769.

15. Yu SL, Singh S, Chen HW, Chen HY, Chen JJ, et al. (2008) Intra-abdominal adhesion formation induces anti-oxidative injury, enhances cell proliferation, and prevents complement-mediated lysis. Wound Repair Regen 16: 388-398.

16. Mutch DM, Simmering R, Donnicola D, Fotopoulos G et al. (2004) Impact of commensal microbiota on murine gastrointestinal tract gene ontologies. Physiol Genomics 16;19(1):22-31.

17. Lecuit M, Sonnenburg JL, Cossart P, Gordon JI (2007) Functional genomic studies of the intestinal response to a foodborne enteropathogen in a humanized gnotobiotic mouse model. J Biol Chem 282: 15065-15072.

18. Mutch DM, Anderle P, Fiaux M, Mansourian R, Vidal K, et al. (2004) Regional variations in ABC transporter expression along the mouse intestinal tract. Physiol Genomics 17: 11-20.

19. Kucherlapati MH, Yang K, Fan K, Kuraguchi M, Sonkin D, et al. (2008) Loss of Rb1 in the gastrointestinal tract of Apc1638N mice promotes tumors of the cecum and proximal colon. Proc Natl Acad Sci U S A 105: 15493-15498.

20. Freilich S, Massingham T, Bhattacharyya S, Ponsting H, Lyons PA, et al. (2005) Relationship between the tissue-specificity of mouse gene expression and the evolutionary origin and function of the proteins. Genome Biol 6: R56.
