# Supplementary material for: Cross-Platform Microarray Meta-Analysis for the Mouse Jejunum Selects Novel Reference Genes with Highly Uniform Levels of Expression
Source: PLoS One. 2013 May 9;8(5):e63125. doi: 10.1371/journal.pone.0063125 (PMC3650031; doi:10.1371/journal.pone.0063125)
Supplement: Table S6 — mRGs stably expressed irrespective of strain, sex, strain by sex interaction and random effect. (DOC) [file pone.0063125.s006.doc]

**Table S6 mRGs stably expressed irrespective of strain, sex, strain by sex interaction and random effect.**

| **Gene^1^** | **Probabilities of significance (*P*) from the linear mixed models analysis of variance for fixed effects** | | |  | **Variance components for random effect** | | |
| --- | --- | --- | --- | --- | --- | --- | --- |
|  | **strain** | **sex** | **interaction of strain and sex** |  | **individual and/ or sample** | **residual error** | **ratio of variance components** |
| ***Plekha7*** | **0.78** | **0.61** | **0.26** |  | 0.45 | 0.58 | **0.78** |
| ***Oaz1*** | **0.31** | **0.28** | **0.88** |  | 0.37 | 0.65 | **0.57** |
| ***Zfx*** | **0.64** | **0.30** | **0.40** |  | 0.03 | 0.03 | **1.04** |
| ***Ube2v1*** | **0.73** | **0.84** | **0.76** |  | 0.45 | 0.64 | **0.71** |
| *Tom1* | **0.48** | **0.78** | **0.54** |  | 0.71 | 0.45 | 1.57 |
| *Hprt1* | **0.91** | **0.49** | **0.57** |  | 0.63 | 0.46 | 1.38 |
| *Tmem14c* | **0.97** | **0.21** | **0.83** |  | 0.85 | 0.23 | 3.61 |
| *B1 element* | **0.17** | **0.82** | **0.76** |  | 0.67 | 0.32 | 2.08 |
| *Hjurp* | **0.61** | **0.78** | **0.76** |  | 0.68 | 0.41 | 1.68 |
| *Cxx1b* | **0.58** | **0.18** | **0.50** |  | 0.64 | 0.48 | 1.33 |
| *D15ertd30e* | 0.0368^c^ | **0.06** | **0.56** |  | 0.26 | 0.48 | **0.55** |
| ***Tspan15*** | **0.59** | **0.67** | **0.56** |  | 0.21 | 0.82 | **0.26** |
| *Zfyve19* | **0.84** | **0.74** | **0.76** |  | 0.79 | 0.34 | 2.36 |
| *AI314976* | **0.64** | **0.48** | **0.80** |  | 0.90 | 0.21 | 4.32 |
| *B2 element* | **0.10** | **0.93** | **0.30** |  | 0.50 | 0.42 | 1.18 |
| *Hadhb* | **0.94** | **0.80** | **0.87** |  | 0.57 | 0.05 | 11.78 |
| *Rps29* | **0.31** | **0.96** | **0.95** |  | 0.80 | 0.27 | 3.03 |
| *Zfp598* | 0.0433^b^ | **0.73** | **0.68** |  | 0.56 | 0.35 | 1.62 |
| *Atp6v0d1* | 0.0110^b^ | **0.25** | **0.56** |  | 0.47 | 0.33 | 1.43 |
| *Pcdha@ / Pcdhc@* | **0.49** | **0.47** | **0.63** |  | 1.06 | 0.18 | 5.83 |
| *Fbln1* | **0.55** | **0.91** | **0.94** |  | 0.76 | 0.35 | 2.18 |
| *Gsr* | **0.12** | **0.74** | **0.75** |  | 0.91 | 0.07 | 12.28 |
| *Aldoa* | 0.0456^(A,B)^ | **0.16** | **0.11** |  | 0.36 | 0.40 | **0.89** |
| *Rpl4* | **0.12** | **0.97** | **0.93** |  | 0.57 | 0.42 | 1.37 |
| *Gag@* | **0.12** | **0.85** | **0.54** |  | 0.88 | 0.09 | 9.32 |
| *Slc52a3* | **0.32** | **0.90** | **0.60** |  | 1.04 | 0.03 | 41.65 |
| *B3gnt* | 0.0057^B,C^ | **0.86** | **0.35** |  | 0.68 | 0.07 | 9.79 |
| *Usmg2* | 0.0045^B^ | **0.51** | **0.62** |  | 0.41 | 0.09 | 4.35 |
| *St6galnac1* | 0.0001^A,b,c^ | **0.67** | **0.46** |  | 0.27 | 0.01 | 18.13 |
| *Fcer2a* | **0.88** | **0.12** | **0.23** |  | 0.95 | 0.01 | 89.11 |

^1^Novel candidate references resulting from meta-analysis protocols I and II restricted to the jejunum (gene symbols in black), classic reference genes (brown), wide-range (more universal) references (green), and SINE elements (grey); @: gene cluster

P values in bold: P >0.05 for all fixed effects, or ratio of variance components below or near 1;

Gene symbols in bold: genes considered as uniformly expressed across samples (P > 0.05 for all fixed effects and ratio of variance components < 1);

Big and small letter superscripts (^A/a^, ^B/b^ and ^C/c^): higher and lower mean expression of a strain, respectively (^A/a^: C57Bl/6 *versus* CD1, ^B/b^: C57Bl/6 *versus* OF1, ^C/c^: CD1 *versus* OF1);

^(A,B)^ mean comparisons significant at P < 0.05 only by the non-adjusted Tukey test (adjusted P values according to the Kramer approximation due to unbalanced data: P = 0.077 and 0.085, respectively).
