# Supplementary material for: Cross-Platform Microarray Meta-Analysis for the Mouse Jejunum Selects Novel Reference Genes with Highly Uniform Levels of Expression
Source: PLoS One. 2013 May 9;8(5):e63125. doi: 10.1371/journal.pone.0063125 (PMC3650031; doi:10.1371/journal.pone.0063125)
Supplement: Table S8 — Details on the RT-qPCR normalization in arbitrarily selected studies of the mouse small intestine. (DOCX) [file pone.0063125.s008.docx]

**Table S8** Details on the RT-qPCR normalization in arbitrarily selected studies of the mouse small intestine.

| **Study issue** | **Reference gene of RT-qPCR** | **Details on validation of normalisation** | **Reference** |
| --- | --- | --- | --- |
| carbonic anhydrase | none | no | [1] |
| regulation of cell fate | cyclophilin* | no | [2] |
| high-glucose diet | *Actb* | no | [[3](#_ENREF_1)] |
| knock-out study | *Tbp* | no | [4] |
| promoter regulation | not applied | no | [5] |
| satiety induction | *Sdtq11*, *Gapdh* | no | [6] |
| fatty acid composition | *Gapdh* | no | [7] |
| stem cells | *Actb* | no | [8] |
| immunological study | *Tbp*, *Gapdh* | no | [9] |
| carboxylesterase expression | *Rn18s* | no | [10] |

*exact gene not specified

**References**

1. Aspatwar, A., Tolvanen, M.E and Parkkila, S. (2010). Phylogeny and expression of carbonic anhydrase-related proteins. *BMC Mol Biol*, 11, 25.

2. D'Errico, I., Salvatore, L, Murzilli, S, Lo Sasso, G, Latorre, D, Martelli, N, Egorova, A.V, Polishuck, R, Madeyski-Bengtson, K, Lelliott, C, Vidal-Puig, A.J, Seibel, P, Villani, G and Moschetta, A. (2011). Peroxisome proliferator-activated receptor-gamma coactivator 1-alpha (PGC1alpha) is a metabolic regulator of intestinal epithelial cell fate. *Proc Natl Acad Sci U S A*, 108, 6603-6608.

3. Du D, Shi YH, Le GW: Microarray analysis of high-glucose diet-induced changes in mRNA expression in jejunums of C57BL/6J mice reveals impairment in digestion, absorption. *Mol Biol Rep* 2010, 37(4):1867-1874.

4. Lussier, C.R., Brial, F, Roy, S.A.B, Langlois, M, Verdu, E.F, Rivard, N, Perreault, N and Boudreau, F. (2010). Loss of hepatocyte-nuclear-factor-1alpha impacts on adult mouse intestinal epithelial cell growth and cell lineages differentiation. *PLoS One*, 5, e12378.

5. Natarajan, K., Xie, Y, Nakanishi, T, Beck, W.T, Bauer, K.S and Ross, D.D. (2011). Identification and characterization of the major alternative promoter regulating Bcrp1/Abcg2 expression in the mouse intestine. *Biochim Biophys Acta*, 1809, 295-305.

6. Naville, D., Duchampt, A, Vigier, M, Oursel, D, Lessire, R, Poirier, H, Niot, I, Bégeot, M, Besnard, P and Mithieux, G. (2012). Link between intestinal CD36 ligand binding and satiety induced by a high protein diet in mice. *PLoS One*, 7, e30686.

7. Nishimoto, T., Pellizzon, M.A, Aihara, M, Stylianou, I.M, Billheimer, J.T, Rothblat, G and Rader, D.J. (2009). Fish oil promotes macrophage reverse cholesterol transport in mice. *Arterioscler Thromb Vasc Biol*, 29, 1502-1508.

8. Stamataki, D., Holder, M, Hodgetts, C, Jeffery, R, Nye, E, Spencer-Dene, B, Winton, D.J and Lewis, J. (2011). Delta1 expression, cell cycle exit, and commitment to a specific secretory fate coincide within a few hours in the mouse intestinal stem cell system. *PLoS One*, 6, e24484.

9. Tetreault, M., Alrabaa, R, McGeehan, M and Katz, J.P. (2012). Krüppel-Like Factor 5 Protects against Murine Colitis and Activates JAK-STAT Signaling In Vivo. *PLoS One*, 7, e38338.

10. Xu, C., Wang, X and Staudinger, J.L. (2009). Regulation of tissue-specific carboxylesterase expression by pregnane x receptor and constitutive androstane receptor. *Drug Metab Dispos*, 37, 1539-1547.
